# Supplementary material for: Implant Fixation and Risk of Prosthetic Joint Infection Following Primary Total Hip Replacement: Meta-Analysis of Observational Cohort and Randomised Intervention Studies
Source: J Clin Med. 2019 May 21;8(5):722. doi: 10.3390/jcm8050722 (PMC6571822; doi:10.3390/jcm8050722)

Supplementary Material

**Supplementary Table S1**	PRISMA checklist
**Supplementary Table S2**	MOOSE checklist
**Supplementary Table S3**	Literature search strategy
**Supplementary Figure S1**	Assessment of risk of bias in randomised controlled trials
**Supplementary Figure S2**	Comparison of all cemented fixation with uncemented fixation and the risk of prosthetic joint infection in observational studies
**Supplementary Figure S3**	Comparison of plain cemented fixations with antibiotic-loaded cemented fixations and the risk of prosthetic joint infection in observational studies
**Supplementary Figure S4**	Comparison of hybrid fixation with uncemented or all cemented fixations and the risk of prosthetic joint infection in observational studies
**Supplementary Figure S5**	Comparison of reverse hybrid fixation with uncemented or all cemented fixations and the risk of prosthetic joint infection in observational studies
**Supplementary Figure S6**	Comparison of all cemented fixation with uncemented or reverse hybrid fixations and the risk of prosthetic joint infection in interventional studies
**Supplementary Figure S7**	Assessment of small study effects by funnel plots and Egger's regression symmetry tests

Supplementary Table S1. PRISMA checklist.

Section/topic	Item No	Checklist item	Reported on page No
		**Title**	
Title	1	Identify the report as a systematic review, meta-analysis, or both	1
		**Abstract**	
Structured summary	2	Provide a structured summary including, as applicable, background, objectives, data sources, study eligibility criteria, participants, interventions, study appraisal and synthesis methods, results, limitations, conclusions and implications of key findings, systematic review registration number	2
		**Introduction**	
Rationale	3	Describe the rationale for the review in the context of what is already known	4–5
Objectives	4	Provide an explicit statement of questions being addressed with reference to participants, interventions, comparisons, outcomes, and study design (PICOS)	5
		**Methods**	
Protocol and registration	5	Indicate if a review protocol exists, if and where it can be accessed (such as web address), and, if available, provide registration information including registration number	2
Eligibility criteria	6	Specify study characteristics (such as PICOS, length of follow-up) and report characteristics (such as years considered, language, publication status) used as criteria for eligibility, giving rationale	6
Information sources	7	Describe all information sources (such as databases with dates of coverage, contact with study authors to identify additional studies) in the search and date last searched	6
Search	8	Present full electronic search strategy for at least one database, including any limits used, such that it could be repeated	Supplementary Table S3
Study selection	9	State the process for selecting studies (that is, screening, eligibility, included in systematic review, and, if applicable, included in the meta-analysis)	6–7
Data collection process	10	Describe method of data extraction from reports (such as piloted forms, independently, in duplicate) and any processes for obtaining and confirming data from investigators	6–7
Data items	11	List and define all variables for which data were sought (such as PICOS, funding sources) and any assumptions and simplifications made	6–7
Risk of bias in individual studies	12	Describe methods used for assessing risk of bias of individual studies (including specification of whether this was done at the study or outcome level), and how this information is to be used in any data synthesis	7–8
Summary measures	13	State the principal summary measures (such as risk ratio, difference in means).	7–8
Synthesis of results	14	Describe the methods of handling data and combining results of studies, if done, including measures of consistency (such as I^2 statistic) for each meta-analysis	7–8
Risk of bias across studies	15	Specify any assessment of risk of bias that may affect the cumulative evidence (such as publication bias, selective reporting within studies)	7–8
Additional analyses	16	Describe methods of additional analyses (such as sensitivity or subgroup analyses, meta-regression), if done, indicating which were pre-specified	7–8
		**Results**	
Study selection	17	Give numbers of studies screened, assessed for eligibility, and included in the review, with reasons for exclusions at each stage, ideally with a flow diagram	8 and Figure 1
Study characteristics	18	For each study, present characteristics for which data were extracted (such as study size, PICOS, follow-up period) and provide the citations	8–9, Table I
Risk of bias within studies	19	Present data on risk of bias of each study and, if available, any outcome-level assessment (see item 12).	9–10, Table I; Supplementary Figure. S1
Results of individual studies	20	For all outcomes considered (benefits or harms), present for each study (a) simple summary data for each intervention group and (b) effect estimates and confidence intervals, ideally with a forest plot	9–10, Figures 2–3
Synthesis of results	21	Present results of each meta-analysis done, including confidence intervals and measures of consistency	9–10, Figure 2; Supplementary Figures. S2–S6
Risk of bias across studies	22	Present results of any assessment of risk of bias across studies (see item 15)	Figure 3
Additional analysis	23	Give results of additional analyses, if done (such as sensitivity or subgroup analyses, meta-regression) (see item 16)	9–10; Figure 3

Section/topic	Item No	Checklist item	Reported on page No
		**Discussion**	
Summary of evidence	24	Summarise the main findings including the strength of evidence for each main outcome; consider their relevance to key groups (such as health care providers, users, and policy makers)	10
Limitations	25	Discuss limitations at study and outcome level (such as risk of bias), and at review level (such as incomplete retrieval of identified research, reporting bias)	13–14
Conclusions	26	Provide a general interpretation of the results in the context of other evidence, and implications for future research	12–14
		**Funding**	
Funding	27	Describe sources of funding for the systematic review and other support (such as supply of data) and role of funders for the systematic review	14

Supplementary Table S2. MOOSE checklist Implant fixation and risk of prosthetic joint infection following primary total hip replacement: meta-analysis of observational cohort and randomised intervention studies.

	Criteria	Brief description of how the criteria were handled in the review
	**Reporting of background**	
√	Problem definition	Prosthetic joint infections (PJIs) though uncommon, are dreaded and devastating complications of total hip replacements (THRs). Whether implant-related factors such as the fixation method influences the risk of infection following THR has been the subject of debate in recent times. In this context, we have carried out a systematic review and meta-analysis to evaluate the body of evidence linking fixation methods (cemented, uncemented, hybrid, or reverse hybrid) with the risk of PJI following THR.
√	Hypothesis statement	Fixation techniques which include cemented, uncemented, hybrid, or reverse hybrid may be associated with the risk of periprosthetic joint infection (PJI) following total hip replacement.
√	Description of study outcomes	Periprosthetic joint infection
√	Type of exposure	Cemented, uncemented, hybrid, and reverse hybrid fixations
√	Type of study designs used	Comparative observational studies and randomised controlled trials
√	Study population	Patients followed for PJI outcomes following total hip replacement
	**Reporting of search strategy should include**	
√	Qualifications of searchers	Setor K. Kunutsor, PhD; Andrew D. Beswick, BSc
√	Search strategy, including time period included in the synthesis and keywords	Time period: from inception to August 2018<br>The detailed search strategy can be found in Supplementary Table S3
√	Databases and registries searched	MEDLINE, EMBASE, Web of Science, and Cochrane databases
√	Search software used, name and version, including special features	OvidSP was used to search EMBASE and MEDLINE<br>EndNote used to manage references
√	Use of hand searching	We searched bibliographies of retrieved papers
√	List of citations located and those excluded, including justifications	Details of the literature search process are outlined in the flow chart. The citation list for excluded studies are available on request.
√	Method of addressing articles published in languages other than English	Not applicable
√	Method of handling abstracts and unpublished studies	Abstracts with no full text publications were not included.
√	Description of any contact with authors	None
	**Reporting of methods should include**	
√	Description of relevance or appropriateness of studies assembled for assessing the hypothesis to be tested	Detailed inclusion and exclusion criteria are described in the Methods section.
√	Rationale for the selection and coding of data	Data extracted from each of the studies were relevant to the population characteristics, study design, exposure, and outcome.
√	Assessment of confounding	We assessed confounding by ranking individual studies on the basis of different adjustment levels and performed sub-group analyses to evaluate differences in the overall estimates according to levels of adjustment.
√	Assessment of study quality, including blinding of quality assessors; stratification or regression on possible predictors of study results	Study quality was assessed based on the nine-star Newcastle–Ottawa Scale using pre-defined criteria namely: population representativeness, comparability (adjustment of confounders), ascertainment of outcome. Sensitivity analyses by several quality indicators such as study size, duration of follow-up, and adjustment factors.
√	Assessment of heterogeneity	Heterogeneity of the studies was quantified with I^2 statistic that provides the relative amount of variance of the summary effect due to the between-study heterogeneity and explored using meta-regression and stratified analyses

√	Description of statistical methods in sufficient detail to be replicated	Description of methods of meta-analyses, sensitivity analyses, meta-regression and assessment of publication bias are detailed in the methods. We performed random effects meta-analysis with Stata 15.
√	Provision of appropriate tables and graphics	Table 1; Figures 1-3; Supplementary Figures S1–S7
	**Reporting of results should include**	
√	Graph summarizing individual study estimates and overall estimate	Supplementary Figures S2–S6
√	Table giving descriptive information for each study included	Table 1
√	Results of sensitivity testing	Sensitivity analysis was conducted to assess the influence of some large studies and low-quality studies on the pooled estimate.
√	Indication of statistical uncertainty of findings	95% confidence intervals were presented with all summary estimates, I^2 values and results of sensitivity analyses
	**Reporting of discussion should include**	
√	Quantitative assessment of bias	Sensitivity analyses indicate heterogeneity in strengths of the association due to most common biases in observational studies. The systematic review is limited in scope, as it involves published data. Individual participant data is needed. Limitations have been discussed.
√	Justification for exclusion	All studies were excluded based on the pre-defined inclusion criteria in methods section.
√	Assessment of quality of included studies	Brief discussion included in 'Methods' section
	**Reporting of conclusions should include**	
√	Consideration of alternative explanations for observed results	Discussion
√	Generalization of the conclusions	Discussed in the context of the results.
√	Guidelines for future research	We recommend nesting analysis within arthroplasty registers as well as definitive randomised controlled trials
√	Disclosure of funding source	In "Acknowledgement" section

Supplementary Table S3. Literature search strategy. Relevant studies, published from inception to 14 April 2019 (date last searched), were identified through electronic searches limited to the English language using MEDLINE, EMBASE, Web of Science, and Cochrane databases. Electronic searches were supplemented by scanning reference lists of articles identified for all relevant studies (including review articles) and by hand searching of relevant journals.

Ovid MEDLINE 1946-Present	
1	exp Hip Prosthesis/ (21604)
2	hip relacement.mp. (0)
3	exp Arthroplasty, Replacement, Hip/ (23365)
4	exp Hip Joint/ (25479)
5	fixation.mp. (195733)
6	cemented.mp. (9687)
7	uncemented.mp. (2634)
8	hybrid.mp. (146856)
9	reverse hybrid.mp. (32)
10	exp Prosthesis-Related Infections/ (10732)
11	periprosthetic joint infection.mp. (900)
12	prosthetic joint infection.mp. (973)
13	prosthetic infection.mp. (396)
14	exp INFECTION/ (739240)
15	exp Surgical Wound Infection/ (33623)
16	surgical site infection.mp. (5502)
17	1 or 2 or 3 or 4 (55289)
18	5 or 6 or 7 or 8 or 9 (350215)
19	10 or 11 or 12 or 13 or 14 or 15 or 16 (741846)
20	17 and 18 and 19 (466)
21	limit 20 to humans (457)
Each part was specifically translated for searching the other databases (EMBASE, Web of Science, and Cochrane databases)	

Supplementary Figure S1. Assessment of risk of bias in randomised controlled trials.

	Randon sequence generation	*Allocation concealment*	*Blinding of participants & personnel*	*Blinding of outcome assessments*	*Incomplete outcome data*	*Selective reporting*	*Other bias*
Wannske, 1979	+	?	-	-	+	?	?
Wykman, 1991	+	?	?	?	+	+	?
Laupacis, 2002	+	+	+	+	+	?	?
Chammout, 2017	+	+	-	?	+	?	?

+	Low risk of bias
?	Unclear risk of bias
-	High risk of bias

Supplementary Figure S2. Comparison of all cemented fixation with uncemented fixation and the risk of prosthetic joint infection in observational studies.

Author, year of publication	No. of patients/hips	No. of PJIs	RR (95% CI)
**All cemented vs Uncemented**			
Dale, 2012	432,168	2,778	1.10 (1.00, 1.20)
Hooper, 2009	42,665	143	1.65 (1.05, 2.60)
Wyatt, 2014	3,319	390	1.28 (1.00, 1.63)
Hailer, 2010	170,413	852	1.11 (0.77, 1.67)
Lenguerrand, 2018	623,253	2,705	1.09 (0.99, 1.20)
Takenaga, 2012	208	4	11.11 (0.61, 203.70)
Pospula, 2008	182	5	4.37 (0.50, 38.33)
Katz, 1992	34	1	2.14 (0.09, 49.08)
Dale, 2011	31,086	236	0.67 (0.45, 1.00)
Dale, 2011	5,540	167	1.00 (0.59, 1.43)
Subtotal			1.10 (1.04, 1.17)
**Antibiotic-loaded cemented vs Uncemented**			
Dale, 2009	97,344	614	0.71 (0.56, 1.00)
Pedersen, 2010	80,756	597	1.24 (0.94, 1.62)
Schrama, 2015	390,671	2,315	1.11 (1.00, 1.25)
Subtotal			1.07 (0.97, 1.18)
**Plain cemented vs Uncemented**			
Pedersen, 2010	80,756	597	1.41 (1.01, 1.96)
Schrama, 2015	390,671	2,315	1.50 (1.20, 1.80)
Engesaeter, 2006	56,275	252	1.80 (1.00, 3.10)
Subtotal			1.50 (1.27, 1.77)

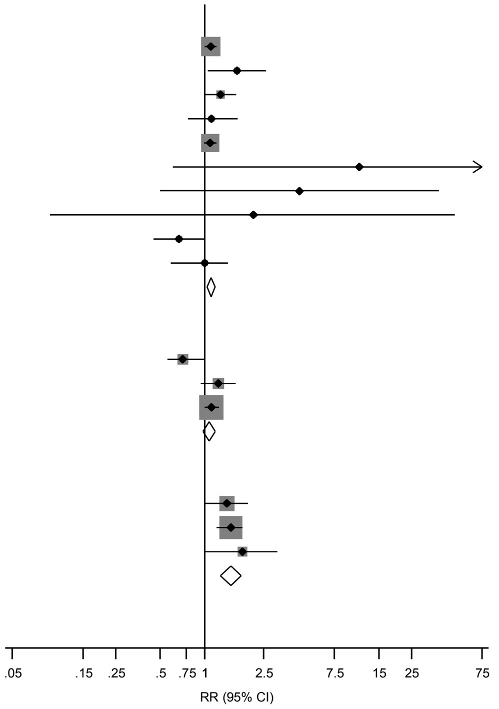

CI, confidence interval (bars); PJI, prosthetic joint infection; RR, relative risk.

Supplementary Figure S3. Comparison of plain cemented fixations with antibiotic-loaded cemented fixations and the risk of prosthetic joint infection in observational studies.

Author, year of publication	No. of patients/hips	No. of PJIs	RR (95% CI)
Dale, 2009	97,344	614	1.90 (1.50, 2.30)
Schrama, 2015	390,671	2,315	1.40 (1.20, 1.60)
Trela-Larsen, 2018	199,205	595	1.36 (0.98, 1.89)
Overall			1.52 (1.36, 1.70)

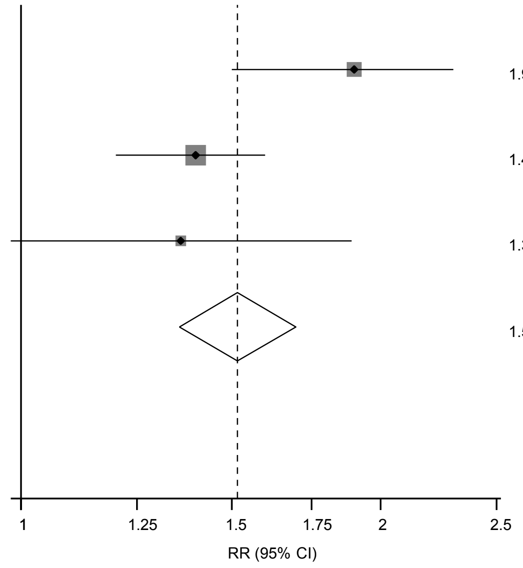

CI, confidence interval (bars); PJI, prosthetic joint infection; RR, relative risk

Supplementary Figure S4. Comparison of hybrid fixation with uncemented or all cemented fixations and the risk of prosthetic joint infection in observational studies.

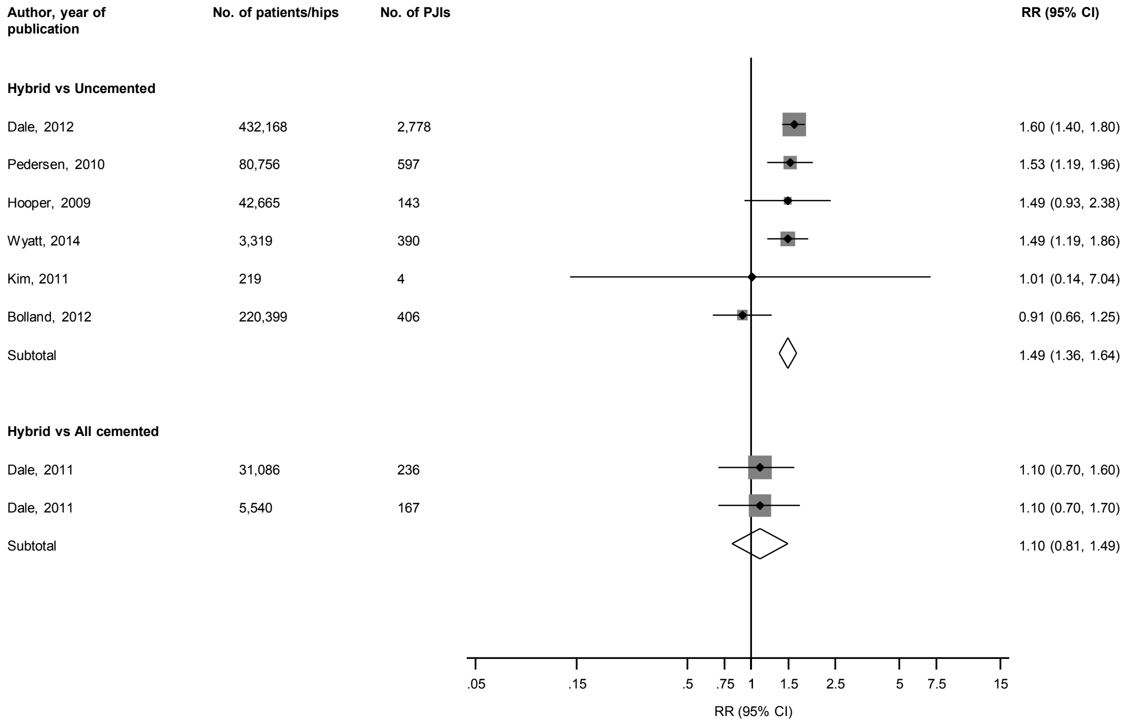

CI, confidence interval (bars); PJI, prosthetic joint infection; RR, relative risk

Supplementary Figure S5. Comparison of reverse hybrid fixation with uncemented or all cemented fixations and the risk of prosthetic joint infection in observational studies.

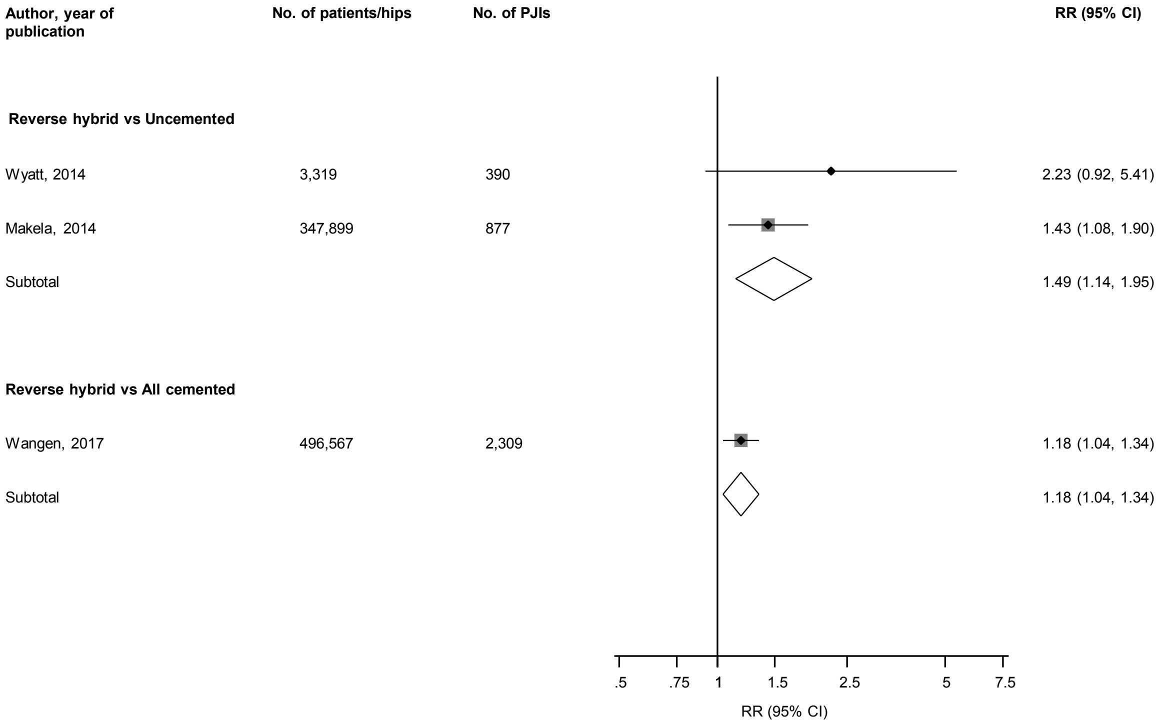

CI, confidence interval (bars); PJI, prosthetic joint infection; RR, relative risk

Supplementary Figure S6. Comparison of fixation types and the risk of prosthetic joint infection in interventional studies.

Author, year of publication	No. of patients/hips	No. of PJIs	RR (95% CI)
**All cemented vs uncemented**			
Wykman, 1991	150	2	5.00 (0.24, 102.43)
Laupacis, 2002	250	2	5.08 (0.25, 104.76)
Subtotal			5.04 (0.59, 42.75)
**Reverse hybrid vs All cemented**			
Chammout, 2017	69	1	3.09 (0.13, 73.22)
**Plain cemented vs Antibiotic-loaded cemented**			
Wannske, 1979	476	15	5.56 (1.56, 20.00)

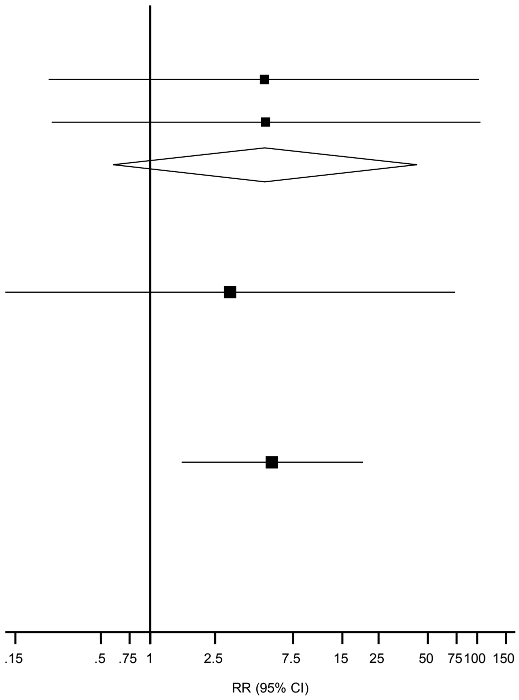

CI, confidence interval (bars); PJI, prosthetic joint infection; RR, relative risk

Supplementary Figure S7. Assessment of small study effects by funnel plots and Egger's regression symmetry tests.

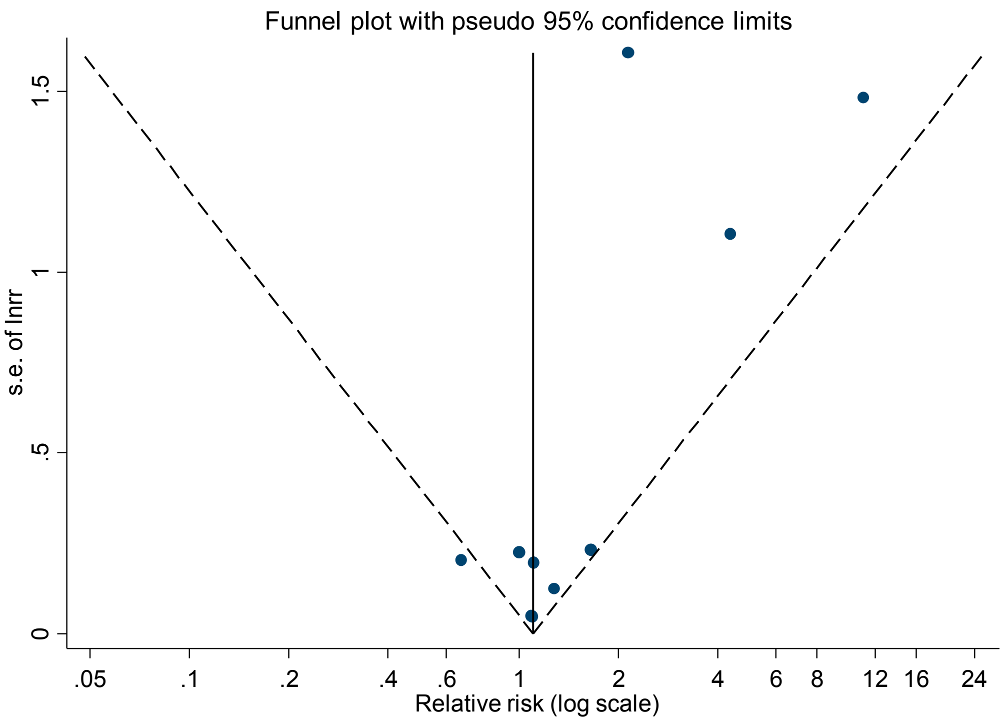

Supplement: Supplementary file 1 [file jcm-08-00722-s001.pdf]
